# Working Right Ways: Investigating Health Practitioners' Perspectives of Foot Health and Needs for Providing Good Foot Care With and for First Nations Peoples

**DOI:** 10.1002/jfa2.70213

**Published:** 2026-10-20

**Authors:** James Gerrard, Shirley Godwin (Badimaya Yamatji), Kim Whiteley (Wiradjuri), James Charles (Kaurna), Vivienne Chuter

**Affiliations:** ^1^ Discipline of Podiatry, School of Health Sciences Western Sydney University Campbelltown New South Wales Australia; ^2^ Wardliparingga Aboriginal Health Equity Unit South Australian Health and Medical Research Institute Adelaide Australia; ^3^ La Trobe University Rural Health School Victoria Australia; ^4^ Independent First Nations Contributor Warramunga–Wiradjuri Country Sydney New South Wales Australia; ^5^ First Peoples Health Unit Griffith University Brisbane Queensland Australia; ^6^ University of Newcastle Gosford New South Wales Australia

**Keywords:** cultural safety, First Nations Peoples (Australia), foot health, participatory action research, practitioner perspective, systemic racism

## Abstract

**Background:**

History of invasion, ongoing colonisation, and systemic racism excluding First Nations Peoples from healthcare are root causes of health inequalities including disparities in foot health outcomes. Sources of inquiry into current practice, learning, and experience, which may shape reform of providing good foot care with and for First Nations Peoples in the land now known as Australia are health practitioners working in the space. This work seeks to explore foot care service delivery by investigating First Nations and non‐Indigenous health practitioners' perspectives regarding providing foot care with and for First Nations Peoples.

**Methods:**

Ways of working are documented in previous work; ‘Working right ways in foot health with and for First Nations Peoples: research method guided and governed by First Nations ways of knowing, being, and doing in cross‐sectional qualitative study design’. First Nations and non‐Indigenous health practitioners provided perspectives with culturally responsive semi‐structured interviews guiding talking with consenting participants. Coding utilised constant comparison techniques to explore patterns across the dataset. Data were analysed using reflexive thematic analysis.

**Results:**

Two talking points engaged health practitioners' perspectives of providing foot care with and for First Nations Peoples: (1) What does foot health mean to you as a First Nations or non‐Indigenous health practitioner? (2) What are needs for providing good foot care with and for First Nations Peoples? Within responses, themes emerged of mobility and participation, overall health and wellbeing, clinical and biomedical perspective, and advocacy being related to the meaning of foot health. The requirements of cultural safety, workforce, and holistic care were perceived as needed for provision of good foot care with and for First Nations Peoples.

**Conclusion:**

Participants described a meaning of foot health that is relational to physical and spiritual constructs, and central to reclamation of, and continuation of, First Nations cultures; amidst a project of ongoing colonisation. Needs for good foot care provision with and for First Nations Peoples included a workforce continually learning and unlearning to develop cultural capabilities to work with and for, and support, First Nations foot health. This study provides distinct and real first‐hand accounts from health practitioners working in the space which the podiatry profession must listen to and receive direction and learning from.

**Abbreviations**
ACCHO: Aboriginal Community Controlled Health Organisation
Ahpra: Australian Health Practitioner Regulation Agency
AMS: Aboriginal Medical Services
HRFS: High Risk Foot Service
IAHA: Indigenous Allied Health Australia
PAD: Peripheral artery disease
PAR: Participatory action research
SEWB: Social and Emotional Wellbeing

## 1 Note

Nomenclature: This work includes the nomenclature; Aboriginal and Torres Strait Islander Peoples, First Nations Peoples, and Indigenous Peoples. Neither singularly nor collectively do they adequately represent the immense diversity of language groups and cultural values across this continent's Traditional Custodians and Sovereign Owners [1, 2]. The authors privilege ways of using terminology that are self‐determined, that communicate diversity and sovereignty, and that minimise use of terms that are imposed. Non‐Indigenous people is the language used to represent and be inclusive of Australians who are not First Nations people [3]. The terms decolonise, decolonisation, and decolonising methodology throughout this work describe being inclusive of First Nations worldviews and holistic conceptualisations of health and wellbeing [4], while challenging and de‐centring dominant colonial views and divesting colonial power [5]. The term method is very much a Western academic term and is described in this publication as ways of working, not just inclusive of, but led by First Nations ways of knowing, being, and doing.

History: The authorship acknowledges that since invasion, Australia's healthcare system has been dismissive of an ongoing and successful First Nations health paradigm in place since time immemorial.

Statistics: When dealing with statistics, pathology, and reported levels of health, authors acknowledge the important considerations and groundings within which to position discussions that respect the strength and pride of First Nations Peoples [6]. The research does not include deficit discourse, does not assign First Nations Peoples as a vulnerable population, and does not present statistics to obscure the root causes of health inequities [6], namely colonisation and systemic racism [6, 7, 8, 9, 10, 11, 12, 13].

Referencing: Referencing follows the Indigenous Archives Collective Indigenous Referencing Guidance for Indigenous Knowledges in acknowledging knowledge creation to address and dismantle oppressive systems denying people creation of their own culture [14]. This work privileges Nation, Country, or Language groups in the reference list, if that information is provided within the source being cited or is clearly provided [14]. This work does not assume a person's affiliation if it is not stated clearly [14].

## 2 Background

Colonisation, everyday discrimination, and racism all negatively impact First Nations Peoples' health and wellbeing [8, 9, 11, 12, 15], with cultural safety deficit in our healthcare system impacting all First Nations Peoples. History of the land now known as Australia is scarred by the exclusion of First Nations Peoples from the conceptualisation, design, implementation, and development of systems including healthcare. History also includes Terra Nullius of intellectual property [16], epistemicide [17], and scientific racism [1] ingraining racism in academia [18] graduating health practitioners. Cultural safety deficit is manifested by racism creating barriers to service access, presenting services as unsafe to approach and use as judged by First Nations Peoples [19].

These systemic issues are reflected in healthcare delivery and outcomes. Within mainstream care delivery, systematic review research describes a lack of culturally safe services in podiatry [20]. This and other barriers to service access contribute to health inequities; First Nations Peoples experience considerably higher rates of diabetes and diabetes‐related foot complications than non‐Indigenous Australians [21], First Nations Peoples experience diabetes‐related foot complications with the mean age up to 14 years younger than non‐Indigenous Australians, and they experience higher risk of peripheral neuropathy, foot ulceration, and amputation [21]. Following adjustment for differences in the age structure of the populations, ‘the rate of hospitalisation associated with diabetes among Indigenous Australians was 4.1 times as high as for non‐Indigenous Australians’ [22]. A state‐wide audit of all amputations performed in Western Australia for years 2000–2008 found that among people 25–49 years of age with diabetes, lower limb major amputations were 38 times more likely and minor amputations 27 times more likely in First Nations Peoples than in non‐Indigenous Australians [23]. These data are especially concerning given that the 5‐year mortality rate following lower limb amputation is second highest to a diagnosis of lung cancer [24].

In response to these inequities, alternative models of care have emerged that prioritise culturally safe services. Across the lands now known as Australia, a self‐determined healthcare system of Aboriginal Community Controlled Health Organisations (ACCHOs) includes provision of foot health services. ACCHOs ‘shift the dynamics of power to centre Aboriginal knowledges, understandings, and perspectives’ [25]. These organisations are primary healthcare services designed and operated by the local Community to deliver culturally safe healthcare for diverse populations across First Nations. ‘ACCHOs came into being because of the inability of mainstream health services to effectively engage Aboriginal Communities with their services’ [26]. Beyond this smaller self‐determined health network though, few foot care services exist for First Nations Peoples, and those that do exist (even within ACCHOs) are rarely evaluated [20]. For mainstream system change, authentic co‐design in foot care service delivery needs to support First Nations‐led, culturally informed, Community‐driven solutions to foot disease. The authentic co‐design of service and governance privileging First Nations knowledges, voices, and worldviews engages First Nations self‐determination in attempting to address mainstream service cultural safety shortfalls including experiences of racism [27].

Alongside Community‐controlled models of care, the importance of culturally safe practice is increasingly recognised. Inclusion of First Nations voices is central to developing culturally safe frameworks across all levels of healthcare design and delivery [28]. Research undertaken on Darkinjung Country describes podiatry services having increased and ongoing Community engagement when culturally responsive aspects feature [29]. Cultural responsiveness involves healthcare provision responsive to traditional and ongoing diverse First Nations cultures and aspects therein [30]. Podiatry service that is led by culturally responsive co‐designed protocols directing care, and that is therefore more likely to be judged as being safe to approach and use, will improve access to support for foot health and assist in reducing diabetes‐related foot complications. This will help reduce hospitalisation due to diabetes‐related foot complications, pursuing health outcome equality for First Nations Peoples. Yet inequities in service delivery and continuing inequality in foot health outcomes remain. Exclusion of First Nations Peoples from health systems design, a past focus on outcomes, an oversupply of aggregated, deficit‐focused, blameworthy statistical data [31], and Western hegemony and scientific racism in research [1, 32, 33] means First Nations Peoples have understandable mistrust of mainstream healthcare [34], while non‐Indigenous healthcare services and practitioners know very little about foot health from First Nations Peoples' perspectives.

The little that is understood in mainstream healthcare about First Nations perspectives of foot and lower limb health is demonstrated by recent systematic review including only four studies [35]. Key outcomes of this research show that firstly, First Nations Peoples include good circulation and sensation in their perceptions of healthy feet and consider access to footwear, and footwear being correctly fitting, as supporting foot health. Secondly, a majority of First Nations Peoples were reported to live with untreated foot pain, with lack of culturally responsive clinicians and culturally safe services perpetuating inequity and inequality in foot health [35]. This limited knowledge is diluted by a Western research process not allowed to know everything about another culture. It is also weakened by systematic review processes likely to exclude knowledges disseminated in ways privileged by First Nations cultures, and knowledges stored in locations that remain unrecognised in mainstream research. This highlights the urgent need for further knowledge of foot health; without extractive colonisation [36], with First Nations leadership, and for First Nations Communities.

In addition to First Nations Peoples' perceptions of their own foot health, First Nations foot health practitioners and clinician allies working in foot health services represent a source of knowledge of current practice, learning, and experience. Understanding the role and importance of foot health to First Nations individuals and Communities and how provision of foot care can support this is essential to reducing the inequities in access to care and inequalities in foot health that continue to shape the reality of outcomes across First Nations. These perspectives can help support future development and shape reform of podiatry in Australia. The aim of this work, therefore, is to privilege First Nations‐led co‐design of ways of working in research to investigate First Nations and non‐Indigenous health practitioners' perspectives of foot health and needs for provision of good foot care with and for First Nations Peoples. Acknowledging that a majority of First Nations Peoples live in regional and urban areas, but also that rural areas are locations of poorer health outcomes for all Australians, including First Nations Peoples [37, 38], this research specifically engaged with practitioners working in regional, rural, and remote areas.

## 3 Methodology

This research is part of a study guided by Indigenous research methodology: Aboriginal Participatory Action Research (PAR) [39]. Work is led by empowered local First Nations leadership, First Nations Reference Group, and First Nations ethical governance. Ways of working privilege First Nations self‐determination.

## 4 Methods

Ways of working in this study are documented in the paper ‘Working right ways in foot health with and for First Nations Peoples: research method guided and governed by First Nations ways of knowing, being, and doing in cross‐sectional qualitative study design’ [40]. In decolonising research [41], authentic First Nations led co‐design of ways of working empowered First Nations self‐determined leadership. Research spaces were anti‐racist places of two‐way learning, which redistributed power to First Nations Peoples. Shifted power dynamic established research spaces that were more likely to be judged as culturally safe for First Nations Peoples to approach and use, as well as work in. Relationships in work were and still are mutually beneficial. Long standing, trusting relationships centred outcomes beneficial for First Nations Peoples as judged by First Nations Peoples.

### 4.1 Study Design and Ethics

Both local First Nations leaders and overarching First Nations governance determined that the frequently used technique within qualitative healthcare research of semi‐structured interviewing [42, 43] would be used for this study. From the outset, ways of working in study design considered how this research may be appraised by the Aboriginal and Torres Strait Islander quality appraisal tool [44] once work was completed. Ethical approval was granted by The Aboriginal Health and Medical Research Council (1376/18) and the University of Newcastle Human Research Ethics Committee (approval No. H‐2018 0035).

### 4.2 Study Participants and Selection Procedures

Semi‐structured interview sample size was *n* = 10, deemed the right mix of participants and enough data to convey a more complicated mosaic of multi‐faceted stories [45, 46, 47].

### 4.3 Participant Recruitment

Sampling that was purposive and seeking large variation was used to recruit health practitioners delivering services relating to foot care with and for First Nations Peoples.

### 4.4 Data Collection

Data collection was conducted online using semi‐structured interviews. Interview guides were developed with First Nations leadership and featured decolonised language. Participants were asked about their perceptions of the meaning of foot health and the needs for providing good foot healthcare with and for First Nations Peoples. First Nations participants were offered having First Nations researcher presence during interviews. Participants consented to interviews while additionally having field notes taken to inform later data analysis. All participants were given the opportunity to verify their data afterwards and First Nations participants were additionally given the opportunity to read the final manuscript drafting prior to any submission for publication. As a part of this process, all four First Nations participants read the final manuscript, combining to offer affirmation for how their data were to be presented in publication as well as some recommendations for the manner in which it was to be discussed. Authors' responses were that minor changes were made to the manuscript to align with participant feedback.

### 4.5 Data Analysis

Ways of working in data analysis included all transcripts being anonymised and transcribed verbatim by researchers manually. Transcriptions and field notes were read, categorised, and inductively coded within the two talking points that guided interviews by an experienced non‐Indigenous researcher who has been working in this space (J.G.) and a First Nations researcher. This partnering brought relational and deeper interpretation to process and minimised cross‐cultural misinterpretation of data in analysis. No software was used to generate the coding framework, which developed from the ongoing discussion of data. Open codes were recorded in Microsoft Word. Data were analysed using reflexive thematic analysis, following Braun and Clarke [48], and coding utilised constant comparison techniques to explore patterns across the dataset. Flexible guidelines presented by Charmaz and Thornberg were utilised as a resource for checking quality in grounded theory [49]. Listening to First Nations voices in analysis, codes were developed both line by line and in larger chunks [49], taking care in disaggregation of data and avoiding any decontextualising. Themes were iteratively developed and refined through ongoing engagement with the data [49, 50, 51, 52]. This included allowance for initial ambiguity, questioning the data with progressive focus, the grouping of codes where themes absorbed or merged with others that is, Mobility and participation, and the presentation of robust connected themes resulting from analysis (Table 2) [49]. All authors agreed upon the thematic analysis, including identifying similarities and differences within and between the interviews and extraction of relevant quotes. Demographic data were managed using Microsoft Excel.

## 5 Results

### 5.1 Foregrounding Results

In privileging First Nations‐led ways of working, it is important to recognise that, when presenting results, depicting discrete themes in tables does not reflect the complexity of the data nor the challenges of this type of analysis and reporting. The story of results of this research describes inter‐connected concepts that are relational to multiple themes and talking points. Themes are not mutually exclusive. Within results relating to the meaning of foot health, the theme of Mobility and participation interconnects with the theme of Overall health and wellbeing for example, describing engaging in physical activities, as well as in cultural business linking to social and emotional state and overall wellbeing. Within results relating to needs for providing good foot care, Cultural safety emerges as a stand‐alone theme. Cultural safety is fundamental to Holistic care for example, where that care needs to be judged by First Nations service users as safe to approach and use, and also connects to Overall health and wellbeing as services require understanding of First Nations ways of belonging. Ways of working in this study acknowledge relationality in the way First Nations stories are told. In the case of First Nations participants, this study recognises voices and knowledges as qualitative data shared by true knowledge holders [53] with authentic lived experiences. First Nations time and contribution to this work are gifts. When presenting results, First Nations data are privileged and are presented first in each topic section of results where it is supported by non‐Indigenous data. Ways of working recognise non‐Indigenous input represents extensive experience and valuable learnings from working with and for First Nations individuals, families, and Communities. This provides context around non‐Indigenous allies' voices; they are valuable and provide some authority in the space for other non‐Indigenous clinicians. Embedding Aboriginal PAR, non‐Indigenous researchers understand the responsibility in taking and interpreting the stories and are led by First Nations advisors. Qualitative data in this study's results tell a story of findings from the work and are presented as such with suffixed ‘First Nations’ or ‘non‐Indigenous’ depending on participant identity.

Demographic data: Ten participants were interviewed in a culturally responsive space. Four participants were First Nations people. Nine participants were podiatrists and one participant worked in wound care involving the foot and lower limb. At the time of interview, participants worked on the lands now known as Western Australia, Northern Territory, Queensland, South Australia, and New South Wales. Participants worked in regional and remote settings across ACCHOs, Aboriginal Medical Services (AMS), hospitals including High Risk Foot Services (HRFS), Community Health Centres, and clinical education settings. Additional demographic information of the included participants has been reported in Table 1. As systemic racism limits the number of First Nations health professionals across the lands now known as Australia [54], including foot‐health professionals, reduced participant characteristic data is published to maintain anonymity. Interview duration ranged from 25 to 63 minutes.

**TABLE 1:** Participant characteristics.

Are you Aboriginal and/or Torres Strait Islander?	How long have you been a registered health professional in your field?	On what Country do you work?	In what setting do you work?	How many First Nations patients do you provide foot healthcare to in terms of overall clientele (responses are as participants described)?
Yes	7 years	Gadigal, Darkinyung and Gumbaynggirr Country	AMS and university	‘Hundreds’
Yes	More than 10 years	Larrakia, Warlpiri, Ngarinyman and Gurindji Country	ACCHO	‘Many communities’
Yes	20 years	Lands of the Yuggera, Turrbal, Yugarabul, Jagera, Yugambeh and Kombumerri Peoples	ACCHO and other clinical and university settings. Aboriginal missions.	‘The majority’
Yes	22 years	Awabakal Country and across many other First Nations	Hospital and community sector. GP practices and aged care	‘Approximately 20% of clientele’
No	4–5 years	Yawuru Country	Outpatient community settings, AMS, aged care and renal	‘The majority, possibly 90% of clientele’
No	13 years	Arrernte Country	ACCHO	‘Predominantly Aboriginal and Torres Strait Islander people’
No	33 years	Kaurna Country and Anangu Pitjantjatjara Yankunytjatjara lands	Almost exclusively ACCHO	‘80%’
No	10–11 years	Darkinjung Country and Wiradjuri Country	ACCHO, Land Council and university	‘All patients are First Nations Peoples’
No	3–4 years	Larrakia Country	High Risk Foot Service, public sector hospital, inpatient and outpatient care	‘60%’
No	5–6 years	Yawuru Country	Public sector with ACCHOs and RFDS	‘80%–90%’

Scope of practice: In terms of foot health and related medical requirements, First Nations and non‐Indigenous participants within this study collectively provided foot and lower limb healthcare with and for First Nations patients which included working in clinics with general practitioners and linking with medical specialists, surgeons, and surgical services. By their own descriptions, participants' scope of foot healthcare practice encompassed delivery of foot health education, diabetes‐related foot care, wound management, preventative care, nail surgery, work with sports and physical musculoskeletal injuries, development of foot health resources, and treatment of high‐risk foot complications (involving neuropathy and peripheral artery disease (PAD), and ulceration and amputation). First Nations and non‐Indigenous participant's work also included provision of education to health professional staff, connection with varied allied health professions (team care including Aboriginal and Torres Strait Islander Health Workers and Practitioners), provision of care in renal units, footwear provision, foot screening, offloading using mechanisms including semi‐compressive felt, Darco shoes, and knee‐high offloading, as well as culturally guided offloading. Responses in data collection described a collective group who additionally support foot health by including family in care and decision‐making, understanding competing priorities in lives, operating Telehealth, flagging escalations in threats to foot health, completing advocacy work, celebrating with people doing really well, and helping support people when they're experiencing barriers to accessing care. Participants were also working to accredit HRFS and working with and for people with more complex medical histories and numerous comorbidities (particularly micro and microvascular changes related to kidney disease, heart conditions, retinopathy, neuropathy, and PAD). Participants also had experience working with and for people in foot health settings whose support overlapped with Mental health teams and Social and Emotional Wellbeing (SEWB) services.

Key overarching quotes relevant to all results and to their interpretation.

I'm not saying these things (building cultural capabilities) are all easy to do, I try to tell, not always young, but often young health professionals about this stuff. But sometimes I don't think they really listen to what we're telling them, and that's part of what we're also saying, we need you to listen

(First Nations)

Priorities in working with First Nations Communities are for non‐Indigenous people to really take time to listen, learn, respect, to build trust, and engage with people

(non‐Indigenous)

We need to be able to practice safely

(non‐Indigenous)

Key themes that emerged from talking points are described in Table 2.

**TABLE 2:** Themes.

Talking points	Themes within talking points
What foot health means to you as a First Nations or non‐Indigenous health practitioner	Mobility and participation
	Overall health and wellbeing
	Clinical and biomedical perspective
	Advocacy
Needs for providing good foot care with and for First Nations Peoples	Cultural safety
	Workforce
	Holistic care

## 6 Themes

### 6.1 Talking Point 1: What Does Foot Health Mean to You as a First Nations or Non‐Indigenous Health Practitioner?

Theme mobility and participation: The theme of mobility and participation emerged regarding First Nations perceptions of the meaning of foot health. ‘Broadly, (foot health) is about sport and trying to keep people active’ (*First Nations*), ‘the obvious, in the sense of people being active and playing sport, then they're less likely to gain weight and those types of things’ (*First Nations*). ‘We've been playing sport for literally thousands and thousands of years and that takes many forms… where the whole Community would play’ (*First Nations*), ‘the importance of that, you often find, you get a lot of, a huge variety of, ages and people playing’ (*First Nations*). First Nations data described a meaning beyond movement connecting to musculoskeletal and cardiovascular health benefits. ‘But it's more about the culture of sport and the importance of being able to participate in that sport. It's more than just sport in a sense, it's culture even’ (*First Nations*), ‘it's more than a sport, so for me to try to help and support people to get back with say… plantar fasciitis or a calf injury or whatever, it might be much more important than, than that' (*First Nations*). ‘There's the participation in cultural events’ (*First Nations*), ‘went out… a couple of weeks ago for a NAIDOC event and, oh, you should have seen it, the whole Community, dancing and participating in the celebrations and, it was just great to see, but, I think that's really important’ (*First Nations*), ‘being able to participate and doing that… is really important, really important stuff’ (*First Nations*).

Non‐Indigenous voice supported the theme of mobility and participation being a perceived element of the meaning of foot health. ‘I think it's just about recognising that our feet are actually amazing because that's what carries us through life, and we need to look after them’ (*non‐Indigenous*). ‘When I think of foot health… my feet carry me through everyday life and take me to all the places that I've been’ (*non‐Indigenous*), ‘I see foot health as the ability to complete the tasks and activities of living that you want to complete’ (*non‐Indigenous*). ‘Personally, I guess foot health is having strong, healthy feet so that I can get around, move, do the things that I want to do, and not have them holding me back from things that I enjoy’ (*non‐Indigenous*), ‘and I think foot health to me really is about looking after my whole body to take me to those places’ (*non‐Indigenous*).

Theme overall health and wellbeing: The theme of overall health and wellbeing being related to the meaning of foot health also emerged from data. ‘To me personally, it's not just about the foot health, it's overall holistic health’ (*First Nations*). ‘I think I'm always trying to think of foot health in relation to much more than the foot’ (*First Nations*), ‘so, foot health to me is probably much broader than the foot’ (*First Nations*). This was again extended beyond physical health by First Nations voices. ‘I think from a lot of different perspectives, and not necessarily only those obvious ones where say diabetes might be having implications for a., b., and c., I think it's broader than that; I think also, social and emotional issues (are part of the meaning of foot health)’ (*First Nations*). ‘And there's a lot more literature now around people's Social and Emotional Wellbeing impact on, let's just say, wound care and wound healing’ (*First Nations*). ‘Even talking… with a lot of really experienced, educated, academics, I still don't feel… I don't know if they really believe that… say someone's Social and Emotional Wellbeing really has those impacts on health’ (*First Nations*). ‘Sometimes I don't feel people really kind of get that’ (*First Nations*).

Data from non‐Indigenous practitioners working in the space supported this theme. ‘Wow! I think it's easy to think about foot health as this segmented part of the body, but I think it's much more than that’ (*non‐Indigenous*). ‘I think foot health is whether your feet and your lower limbs are in good health or not, but I think it's very much related to whole‐body health’ (*non‐Indigenous*). ‘I think it (foot health) also has implications for how you can function or exist or live within society and within your family as well—because of the systemic effects that it (loss of foot health) can have’ (*non‐Indigenous*).

Theme clinical and biomedical perspective: A theme with more clinical biomedical perspective associated with the meaning of foot health also emerged. ‘As a podiatrist, my role is to try and encourage healthy feet and to try and enable people to be able to complete their activities of daily living as best they can’ (*non‐Indigenous*), ‘so I try my best to provide that sort of treatment and preventative care to clients’ (*non‐Indigenous*). ‘I guess to me, foot health means being aware of the risks associated (to foot health when living with diabetes)… doing all the things that you can do; checks, seeing a podiatrist regularly, even educating other friends or family on things that they can do’ (*non‐Indigenous*).

Theme advocacy: First Nations voices established a theme of advocacy, describing that, despite progress in the space, the meaning of foot health includes representation of the impacts of ongoing colonisation and health inequities. ‘There is a lot of work still to do, but I think there are also a lot of really encouraging signs, it's something that is increasingly being put on the radar by a number of really great podiatrists and researchers, and also Communities themselves, chronic illness as a whole is something that the Aboriginal Community in the Aboriginal Medical Service space is continuing to draw focus to… and within that space, issues relating to diabetes and the impact that diabetes has on the lower limb are increasingly getting the notice and attention of the Community and the Aboriginal Medical Service Community’ (*First Nations*). However, ‘I'm very conscious about the debilitating impact that poor foot health can have on Aboriginal people and the Community more broadly, and you know, the contribution that it makes to this day of a really unfortunate trauma that's very common among our people—I have a conversation with any Aboriginal person, they will, I imagine, have personal family experience with diabetes and that experience will increasingly involve an end stage intervention in the lower limb, be that an amputation of some variety, or a wound with wound care of some variety, and I'm very conscious that you know intervening, educating to prevent, that's what foot health means to me’ (*First Nations*). First Nations voices related current foot health inequity with systems underpinning practice. ‘It's ‘not just the face‐to‐face podiatric care that you might get, but all the systems around actually getting those foot services’ (*First Nations*). ‘When I went to university… I felt like, well, when I started practicing, I realised there was a big gap between what you get to in a university and then what you might see in a remote Community and how you might treat Aboriginal and Torres Strait Islander people in a remote Community’ (*First Nations*), ‘everything from presentations to support services; or more services that might be available to provide support to you if you're providing care in a remote Aboriginal Torres Strait Islander Community’ (*First Nations*). ‘Education and training and mainstream resources are tailored around a continued access to podiatric services… for clients and Communities (living) with diabetes… which isn't always the case in remote Communities’ (*First Nations*). First Nations voices included self‐reflection within the meaning of foot health: ‘As an Aboriginal person… it is crucial that I am aware of looking after my own health’ (*First Nations*), and spoke to self‐determination, ‘so, how you look after your own health, your own needs’ (*First Nations*).

Non‐Indigenous voices also spoke to the theme of advocacy, projecting learnings of non‐Indigenous clinicians working and developing skills in the space. ‘Foot health to me really means enhancing and maintaining mobility and independence and ability to be in (First Nations) Community and with (First Nations) Community more than anything else’ (*non‐Indigenous*). ‘So, it's about the ideal in foot health—that people ignore it because there isn't a problem rather than ignore it because there is a problem’ (*non‐Indigenous*). ‘It's having that ability for (First Nations) Communities to have functional and operational ability to mobilise without risk of injury’ (*non‐Indigenous*), and ‘it's… the awareness of and being empowered to make decisions’ (*non‐Indigenous*).

### 6.2 Talking Point 2: Needs for Providing Good Foot Care With and for First Nations Peoples

Theme cultural safety: The theme emerged of the need for providing good foot care with and for First Nations Peoples to be safe, as judged by First Nations Peoples themselves. Thematic analysis described the foot care model most common in Australia as one which is ‘highly institutionalised’ (*First Nations*) that ‘isn't necessarily something that is conducive to uptake in the Aboriginal Community’ (*First Nations*). A need for service delivery ‘much less formal in nature but not at all ineffective for not having that very institutional focus’ (*First Nations*) exists because when First Nations Peoples are required to interact with a high‐risk foot clinic, for example, ‘there is a hesitation there that can be quite difficult to overcome’ (*First Nations*).

Regarding safer service provision, ‘so much is to go to provide service to people in their home or their home Community… where they feel comfortable… and not so out of place, more willing to perhaps ask questions, which is important for success, for healing… and for empowerment of the person receiving the care’ (*First Nations*). ‘I think if there's a government run, or an organisation that is wanting to, for very good reason, provide care, they need to do it in an appropriate way’ (*First Nations*).

Non‐Indigenous data supported the emerging theme. Foot care service is often embedded in a ‘large system where they're quite directive about what will and won't happen’ (*non‐Indigenous*). ‘Historical racism, injustices, ways that the health service has been structured, has disempowered and disadvantaged First Nations people; and so, I think we've got a long way to go in providing a respectful health service’ (*non‐Indigenous*). The emerging need for safe service highlighted ‘different needs depending on place and Community’ (*non‐Indigenous*) including the ‘structure of the clinic so it's run and set up in a very friendly… culturally safe way’ (*non‐Indigenous*). Results described the ‘length of appointments’ and ‘the idea of time’ being variable, instances of ‘not needing appointment times’ (*non‐Indigenous*), and instances of ‘communal consultation’ (*non‐Indigenous*) where people ‘can come and just have a chat, talk to other members of the Community, talk with Elders, and talk with us (health professionals)’ (*non‐Indigenous*). This theme of safe structure of foot care clinics included ‘being co‐designed by Community, led by Community, and evaluated by Community and the Elders’ (*non‐Indigenous*). ‘I think that tries to increase engagement with an uptake of the service and in doing so, hopefully, provides an opportunity to provide culturally safe, which would have to be deemed by First Nations people, care to (people) to address their foot health needs as best as possible’ (*non‐Indigenous*).

Adjunct to this theme, analysis of First Nations data highlighted needs for education resources and staff underpinning a safe foot care environment to be culturally responsive. ‘It's been hard, it was hard to find resources around education and supporting self‐care… or education around how to provide health care, to go and get foot care, that was appropriate for Aboriginal and Torres Strait Islander people’ (*First Nations*) as judged by them. Non‐Indigenous voices added, ‘you've got people (non‐Indigenous workforce) culturally just not taking the time to acknowledge that every group and every Community are different’ (*non‐Indigenous*). ‘There might be family or people in (foot health settings) where they (First Nations Peoples) can't go in at the same time’ (*non‐Indigenous*); First Nations Peoples ‘might associate the clinic with sick or poor health’ (*non‐Indigenous*). ‘I just noticed the huge difference between say the saltwater people and the freshwater people and adapting the service between different cultural groups, and different language groups. It's a challenge, but it's really, it's really cool to actually reflect back and see the difference’ (*non‐Indigenous*). ‘And I think being flexible in where the service is provided is essential in being able to provide good health education and services to everyone. And I also think as well, the type of education that is provided has to be unique to everyone.’ (*non‐Indigenous*).

Theme workforce: The theme of workforce emerged from data analysis, essentially being a need for a sustained workforce to provide continued podiatry services with and for First Nations Peoples. Need for funding underpinned this, identified as being required for travel and more podiatrists, podiatrists with ever developing cultural capabilities. Highlighted workforce needs were ‘people (health professionals) who are patient’ (*First Nations*). ‘Listening, I think is really, really important… (health professionals) listen to what people are telling them and… look for the body language and those other cues… that are often important in Aboriginal cultures’ (*First Nations*). This extended to the need for workforce to be culturally humble, and in doing so to position themselves as learners in cross cultural experiences to reposition power within clinical environments. ‘I think one of the other important needs is humility’ (*First Nations*). ‘I think people need; they need providers to be more humble’ (*First Nations*). ‘People (health professionals) have the right to be kind of proud of their own success in their careers… but I think they need to listen as well’ (*First Nations*). ‘They need to understand that they're not the be all and end all. They don't know everything they need to’ (*First Nations*). Non‐Indigenous voices supported this theme, highlighting workforce ‘using time for talking’ (*non‐Indigenous*). ‘The time to sit down and create a relationship and build some trust with patients is really important’ (*non‐Indigenous*), ‘it takes commitment to be able to develop that trust within Community.’ ‘And not just to go and get what you want out of it as, as often is the case with Western healthcare or research’ (*non‐Indigenous*). Non‐Indigenous voices supported cultural humility in workforce, with the importance of Community ‘having that sense of control that it is the individuals’ and the Community's right to determine what is important and what's not, and what needs to be done’ (*non‐Indigenous*). ‘People (recipients of care) are marginalised by systems because everybody (health systems) tells everybody (service users) what they should be doing and what's good for them, rather than actually asking the individual what's important to them’ (*non‐Indigenous*). ‘People get talked at rather than talked with, or there is no sitting down and getting that two‐way conversation’ (*non‐Indigenous*). The need for culturally capable health professionals developing ‘two‐way conversation… that allows people to go away and make their own decisions and to decide their own priorities’ (*non‐Indigenous*) and ‘two‐way respect for everybody's perspective of what's important’ (*non‐Indigenous*) were identified. In addition to a larger and progressively culturally capable non‐Indigenous workforce, First Nations workforce was seen as invaluable. ‘I think Aboriginal staff in the region that I'm working in are probably (the service's) biggest asset, (First Nations staff) can help with communication, breaking down cultural barriers… following those cultural and family links and knowing when and where people are around, and when they can come into the clinic and when they can't—all those things are really important’ (*non‐Indigenous*).

Theme holistic care: The theme emerged of overall health being a need for and being directly related to providing good foot healthcare with and for First Nations Peoples. Results included ‘diet… eating well… sustaining life… and protecting yourself… checking your feet’ (*First Nations*), for example. Hub clinic resources reaching regional and remote Communities, Telehealth services, and education were identified as needs for holistic care provision and this encompassed providing holistic foot care which centred culture; ‘I think they (health professionals) really need to be open and be able… to provide a full service, have an understanding of culture, the culture of the place where they're going, and that's always different—there might be some similarities, but it might be very different from another’ (*First Nations*).

Supportively, non‐Indigenous data added ‘empowerment and self‐determination is definitely a need’ (*non‐Indigenous*), and ‘empowering Indigenous people to make health decisions for themselves’ (*non‐Indigenous*) was found to be foundational to holistic care.

## 7 Discussion

Many interconnected elements—the meaning of foot health: The meaning of foot health was described by First Nations practitioners in this study as associated with aspects of culture and Community, particularly the importance of mobility in continuing cultural practices. First Nations participants defined foot health far beyond being able to physically ambulate and complete individual‐focused tasks associated with daily living. This included active participation in inclusive sports, in cultural events, and in Community celebrations, which were identified as particularly important. Non‐Indigenous voices related the meaning of foot health to mobility more generally. These findings speak to broader concepts of health and wellbeing. First Nations health is ‘not just the physical wellbeing of an individual but refers to the social, emotional and cultural wellbeing of the whole Community in which each individual is able to achieve their full potential as a human being thereby bringing about the total wellbeing of their Community’ [55, 56], and ‘Land is central to wellbeing’ [39]. Gee et al., 2014 conceptualise one's Social and Emotional Wellbeing from a First Nations perspective as being inseparable from Community and connected to culture, family and kinship, body, spirituality and Ancestors, and to Country [57]. This points to there being many more and much deeper aspects to the meaning of foot health than are currently taught and understood by Western academia and biomedical healthcare across the lands now known as Australia. For example, the impacts of diabetes‐related foot disease can extend far beyond direct physical injury. A fourfold increased risk of peripheral neuropathy [58] results in First Nations Peoples not being able to feel Country and connect with Country through their feet, in ways they were able to prior to pathology. Altered footprints post digital amputation means that Country may no longer recognise the person who made those footprints [59]. These examples only provide a glimpse into the value of foot health and its meaning to First Nations Peoples. The contribution of foot health to rebuilding ways of celebrating, storing knowledges, keeping people healthy, and maintaining cultural practices places the meaning of foot health as being critical to achieving and maintaining overall health around which culture is centred [57, 60]. The 2021 Australian evidence‐based guidelines for diabetes‐related foot disease [61] demonstrate that there is growing recognition of the importance of feet to culture and Country that has previously been absent from mainstream foot care provision. For the first time, international guidelines contextualised to working in Australia included First Nations experts inputting First Nations knowledges and worldviews at its heart [61], led by Professor James Charles (Kaurna). Through Professor Alex Brown (Yuin), these guidelines urge readers and users to engage with First Nations Peoples and First Nations controlled health services ‘in the pursuit of health equity’ [61]. Foot health being acknowledged as critical to connection with Country, Community, and culture is essential to the reclamation of practices connecting to entities ignored, illegalised, dehumanised, removed, stolen, and outlawed by invasion and ongoing colonisation [15].

Within this study, First Nations perspectives identified meaning of foot health as inter‐relational with SEWB; as being a part of overall health, but also as being influenced by social and emotional issues, for example, wound healing. Mainstream literature reports psychological stress having statistically significant and clinically relevant impacts on wound healing [62], which is consistent with First Nations perspectives from this study. In terms of psychological stressors impacting overall health, systematic review and meta‐analysis of data globally found the stressor racism associated with both poorer general and physical health [63]. In a national context, systematic scoping review has associated racism with negative overall mental and general health outcomes for First Nations Peoples living in the land now known as Australia [9]. Working and learning in spaces supporting First Nations foot health, non‐Indigenous participants' perspectives in this present study contributed to the theme of the meaning of foot health being connected to ‘Overall health and wellbeing’ and included systemic impacts, whole body, and family. To be highlighted, some First Nations participants raised perspectives of doubt in experienced academics working in the foot health space being understanding of SEWB impacts on health. This is concerning given their research and teaching informs clinical service delivery [64]. First Nations perspectives also included the impacts of colonisation and health inequities as core to the meaning of foot health, identifying poorer foot health as contributing to ongoing trauma experienced by First Nations Peoples. To achieve equity in foot health for First Nations Peoples, non‐Indigenous people in all foot health‐related spaces, including experienced academics [64], must position themselves as learners and engage deeper listening [65] to better understand a meaning of foot health informed by First Nations knowledges and perspectives. A meaning including, but not limited to, spiritual and ancestral relationality, SEWB, connection to Country and place, remaining part of knowledge disseminating storylines, and strength and pride in keeping feet strong as a marker of health and wellbeing in the face of ongoing colonial systemic racism.

Cultural safety leading service design: Overwhelmingly, First Nations and non‐Indigenous health practitioners perceived foot care services provided with and for First Nations Peoples as needing to be culturally safe, as judged by service users. As an essential part of culturally safe foot healthcare, the need to recognise and understand the existence of racism (individual and systemic) in foot care provision with and for First Nations Peoples more widely, was identified in this work by both First Nations and non‐Indigenous data. Racism's negative impacts on First Nations health is well documented in literature [9]. In recognition of this the Australian Health Practitioner Regulation Agency (Ahpra) mandates ‘health practitioners must acknowledge… systemic racism, … and provide care that is holistic, free of bias and racism’ [19]. Anti‐racism in health service provision including foot care service is therefore not an option but it too is a legal requirement [66].

With this in mind, First Nations and non‐Indigenous practitioners in this study identified an institutionalised healthcare model as predominant in Australia, contributing to foot care provision being largely judged by First Nations Peoples as unsafe to approach and use. As a tool of colonisation of Australia, First Nations exclusion from healthcare paradigms is well documented [15, 67] and has resulted in the establishment of a mainstream healthcare system which is fundamentally unsafe for First Nations Peoples. Findings of this study implicate institutionalised foot health systems in disparities between health outcomes today. Current inequalities of Australia's healthcare system are an 8–9 year less life expectancy for First Nations Peoples [68, 69], First Nations burden of disease 2.3 times the rate of non‐Indigenous Australians, and burden of diabetes 6 times as high [70]. Ahpra partners with national health regulatory boards to safeguard the public when accessing healthcare, ensuring registered health practitioners are qualified and safe [71]. It is mandated by Ahpra that ‘health practitioners must acknowledge colonisation’ [19]. In this study, First Nations and non‐Indigenous perceptions of the need for foot care services to be delivered outside of institutionalised settings and, where possible, on Country specify cultural safety improvements to foot care services linking colonisation to Country [15, 57, 60, 72, 73]. This respects the sorrow and trauma of loss of Country through invasion [15, 74], and acknowledges the ongoing denial of access to Country being a human rights issue [75, 76, 77]; both of which impact health and SEWB [78], and disrupt First Nations cultural wellbeing [57]. Existing literature documents brutal historical institutionalised healthcare provision associated with hospitals, just one example being the forcible removal of First Nations people from Country to medical incarceration in Lock Hospitals [79, 80, 81]. Transitioning to foot health service provision on Country represents an example acknowledging and addressing the harm caused by colonised healthcare; foot care on Country is inclusive of a protective health factor for First Nations Peoples, centres cultural determinants of health [60], and provides more culturally responsive service [30].

Culturally responsive service delivery: This study's findings and discussion are about action and developing skillsets for non‐Indigenous individuals to change non‐Indigenous foot health systems that work best only for non‐Indigenous people. In this study, participants working inside and outside of Community‐controlled services provided First Nations and non‐Indigenous health practitioner perspective of clinicians being able to address mainstream cultural safety deficit by improving deeper listening [65] and cultural capabilities. This needs to happen via ongoing anti‐racism and cultural capability learning on a personal level across foot health clinical settings, especially in positions of power. Such action seeks to provide positive foot healthcare experiences for First Nations Peoples. The findings presented in this work identify that there are historical and ongoing systemic discriminatory factors which need addressing in delivering foot care services with and for First Nations Peoples, that go beyond the Western biomedical theory and clinical skills privileged in teaching institutions, ingrained in graduates, and prioritised by clinicians in the land now known as Australia.

Ongoing research story: This study contributes to existing work and commentary in the field of cultural safety and First Nations foot health research [19, 20, 29, 82, 83]. Our aim was for this work to bring ways of being and doing in foot health that can make spaces safer for First Nations Peoples to work in, as judged by them, and that can produce First Nations‐led ways of knowing to improving, changing, and informing inclusive and responsive foot healthcare delivery. Participants' perspectives align with broader reported First Nations health lived experience, guidelines, plans, strategies, and published works [19, 20, 30, 84, 85]. This study provides qualified, voiced lived experiences which the podiatry profession must listen to and receive direction from.

## 8 Strengths and Limitations

Strengths of this research are embedded in the aims to bring benefits to First Nations Peoples. This work addresses needs as identified by First Nations Peoples, empowers First Nations leadership, privileges First Nations voices, and seeks First Nations evaluation in ways of working with and for. It also brings attention to perceived needs for providing good foot care with and for First Nations Peoples. In doing so, the results illustrate ways of working to address inequities in foot health service delivery, which continue to contribute to inequality in health outcomes. A limitation of the work is that it includes concepts that are beyond the scope of research to discuss in full; discussion is confined to the parameters, formatting, and author guidelines of publication. Although many themes exist within each of the two talking points, they are certainly not mutually exclusive, and it is not the intention of this work to oversimplify relationality or to minimise interconnectedness of complex constructs. Additionally, working online can affect participant access to taking part in semi‐structured interviews as well as the richness of data collection; however, First Nations Advisory Group leading ways of working in research did not see that presenting as a limitation in this study. Whilst a strength of the study may be having 9 out of 10 participants being podiatrists, conversely a potential limitation is that the voices of other foot health workers such as Aboriginal Health Practitioners and General Practitioners are missing. Given the diversity in regions and settings within which participants worked and have worked however, the application of findings across professions, organisations, and settings is considered to be transferable in partnership with transformative learning. Be this ways of working in research, ways of doing in clinical practice, or ways of teaching in foot care education, findings from this study can develop cultural responsiveness understandings, which can be actioned and embedded throughout foot care praxis. A limitation related to this though, is the research being based in what mainstream foot care services historically wouldn't promote as required ongoing continuing professional development for podiatrists. When reading and considering this work, this authorship group urge self‐reflexivity: to read further where learning is needed, to improve skills, and to advocate for ongoing development of individual and foot care systems' cultural responsiveness.

## 9 Conclusion

This work followed authentic First Nations‐led co‐design in ways of working in research to evaluate First Nations and non‐Indigenous foot health practitioners' perspectives of foot health and needs for provision of good foot care with and for First Nations Peoples. This study found the meaning of foot health related to mobility and participation facilitating overall health and SEWB. The study also found that the meaning of foot health is relational to physical and spiritual constructs, and that it is central to reclamation of and continuation of First Nations cultures amidst a project of ongoing colonisation. In this research, the needs for good foot care provision with and for First Nations Peoples included cultural safety, as judged by First Nations Peoples, delivery of foot healthcare centred on Country and outside of institutionalised settings, and foot healthcare supported by a workforce continually learning and unlearning to develop cultural capabilities to work with and for, and support, First Nations foot health. Practical implications of this study are foot health service designs changing to become ever more culturally responsive: to places, to people, and to providing positive foot healthcare experiences. This study provides informed perspectives of health practitioners working in the space to support ongoing efforts to do better in this space. Further work needs to privilege the voices and perspectives of First Nations individuals, families, and Communities in leading working with and for them to keep feet strong. To have greatest impacts and to privilege authentic co‐design, research needs to occur in and with and for the First Nations Communities who need and want to establish and improve foot health services to work better; with and for them as users of services.

## Author Contributions

**James Gerrard:** project administration, data curation, formal analysis, writing – original draft, writing – review and editing. **Shirley Godwin (Badimaya Yamatji):** supervision, validation, data curation, formal analysis, visualization, methodology, writing – review and editing. **Kim Whiteley (Wiradjuri):** supervision, validation, formal analysis, visualization, methodology. **James Charles (Kaurna):** supervision, validation, formal analysis, visualization, methodology. **Vivienne Chuter:** supervision, data curation, formal analysis, visualization, validation, methodology, writing – review and editing.

## Funding

The authors have nothing to report.

## Ethics Statement

Ethical approval was granted by The Aboriginal Health and Medical Research Council (1376/18) and the University of Newcastle's Human Research Ethics Committee (approval No. H‐2018 0035).

## Consent

The culturally responsive Participant Information Statement received by all participants included information that de‐identified findings would be used in a publication. All participants provided written informed consent.

## Conflicts of Interest

The authors declare no conflicts of interest.

## Supporting information


Supporting Information S1

Supporting Information S2

## Data Availability

The datasets generated and/or analysed during the current study are not publicly available. Aligning with Maiam nayri Wingara principles of Indigenous Data Sovereignty and Governance, they are available from the corresponding author on reasonable request. The Aboriginal and Torres Strait Islander Quality Appraisal Tool is included as a supplementary file.
