# Supplementary material for: Working Right Ways: Investigating Health Practitioners' Perspectives of Foot Health and Needs for Providing Good Foot Care With and for First Nations Peoples
Source: J Foot Ankle Res. 2026 Oct 20;19(4):e70213. doi: 10.1002/jfa2.70213 (PMC13683549; doi:10.1002/jfa2.70213)
Supplement: Supplementary file 2 [file JFA2-19-e70213-s002.pdf]

ABORIGINAL AND TORRES STRAIT ISLANDER QUALITY APPRAISAL TOOL

CREATE

Answer either "Yes", "Partially", "No" or "Unclear" to each question

Article citation: Working right ways: Investigating health practitioners' perspectives of foot health and needs for providing good foot care with and for First Nations Peoples   Date: 18 August 2026

Reviewer's name: Article authors: Dr Shirley Godwin (Badimaya Yamatji) and James Gerrard

Question	Yes	Partially	No	Unclear
1. Did the research respond to a need or priority determined by the community?	☑	☐	☐	☐
2. Was community consultation and engagement appropriately inclusive?	☑	☐	☐	☐
3. Did the research have Aboriginal and Torres Strait Islander research leadership?	☑	☐	☐	☐
4. Did the research have Aboriginal and Torres Strait Islander governance?	☑	☐	☐	☐
5. Were local community protocols respected and followed?	☑	☐	☐	☐
6. Did the researchers negotiate agreements in regards to rights of access to Aboriginal and Torres Strait Islander peoples' existing intellectual and cultural property?<br>This research is part of a PhD with four First Nations people registered as supervisors. This leadership oversees and maintains Maiam nayri Wingara principles principles of Indigenous Data Sovereignty and Indigenous Data Governance with and for all First Nations particpants in work.	☑	☐	☐	☐
7. Did the researchers negotiate agreements to protect Aboriginal and Torres Strait Islander peoples' ownership of intellectual and cultural property created through the research?<br>This research is part of a PhD with four First Nations people registered as supervisors. This leadership oversees and maintains Maiam nayri Wingara principles principles of Indigenous Data Sovereignty and Indigenous Data Governance with and for all First Nations particpants in work.	☑	☐	☐	☐
8. Did Aboriginal and Torres Strait Islander peoples and communities have control over the collection and management of research materials?<br>This study is part of a PhD project in which First Nations Communities, participants, and Country hold control of the data they provide as research participants. Culturally responsive consent and information forms developed with First Nations leadership were and still are specific to this study and explicitly not for extended or unspecified uses.	☑	☐	☐	☐
9. Was the research guided by an Indigenous research paradigm?	☑	☐	☐	☐
10. Does the research take a strengths-based approach, acknowledging and moving beyond practices that have harmed Aboriginal and Torres Strait peoples in the past?	☑	☐	☐	☐
11. Did the researchers plan and translate the findings into sustainable changes in policy and/or practice?<br>This study is part of a PhD project with overarching First Nations-led knowledge-translation plan. Implementation includes publication of study manuscripts in Journals; part means to develop foot health curricula and inform praxis.	☑	☐	☐	☐
12. Did the research benefit the participants and Aboriginal and Torres Strait Islander communities?	☑	☐	☐	☐
13. Did the research demonstrate capacity strengthening for Aboriginal and Torres Strait Islander individuals?<br>Whilst no First Nations Peoples or organisations were directly employed or trained as a part of work, First Nations Peoples gained experience in leading and conducting research which does strengthen professional capacity.	☐	☑	☐	☐
14. Did everyone involved in the research have opportunities to learn from each other?	☑	☐	☐	☐
